# Plasmid-mediated Quinolone Resistance in *Salmonella enterica*, United Kingdom

**DOI:** 10.3201/eid1402.070573

**Published:** 2008-02

**Authors:** Katie L. Hopkins, Martin Day, E. John Threlfall

**Affiliations:** * Health Protection Agency Centre for Infections, London, United Kingdom

**Keywords:** Salmonella enterica, variable number of tandem repeats, antimicrobial drug resistance, plasmids, letter

**To the Editor:** Fluoroquinolones are broad-spectrum antimicrobial drugs used to treat many clinical infections. Salmonellosis is treated with fluoroquinolones only in elderly or immunocompromised patients, but these drugs are also used for treating patients with enteric fever, invasive disease, or long-term salmonellae carriage. High-level fluoroquinolone resistance is uncommon, but reduced susceptibility is increasing.

Since 1998, plasmid-mediated quinolone resistance encoded by *qnr* genes A, B, and S that confer low-level resistance to nalidixic acid and reduced susceptibility to ciprofloxacin has been identified in several enterobacterial species, including *Salmonella*. Their clinical importance is in facilitating resistance to potentially lethal levels of quinolone. Additionally, *qnr* genes are often associated with strains that produce extended-spectrum β-lactamases.

We recently reported identification of *qnr* genes in *Salmonella* in the United Kingdom (*1*). Most isolates were associated with the Far East. Two isolates of *S*. Virchow were part of an outbreak associated with imported cooked chicken from Thailand. During October 2006–April 2007, we monitored *qnr* genes in nontyphoidal salmonellae isolated in the United Kingdom that expressed reduced susceptibility to ciprofloxacin (MIC 0.125–1.0 μg/mL) with concomitant susceptibility to nalidixic acid (MIC <16 μg/mL). This resistance phenotype is a useful marker for the *qnr* gene as the sole quinolone resistance determinant (*1*).

Recent studies showed that isolates of *Salmonella* spp. and *Escherichia coli* with decreased susceptibility to ciprofloxacin (MICs >0.06 μg/mL and 0.5 μg/mL, respectively), but with susceptibility or intermediate resistance to nalidixic acid (MIC 8–16 μg/mL and 4–8 μg/mL, respectively), all had *qnrA* or *qnrS* genes but lacked mutations in the topoisomerase genes (*2**,**3*). Strains with ciprofloxacin MICs >1 μg/mL were also included to monitor involvement of *qnr* genes in development of high-level ciprofloxacin resistance. Breakpoint concentrations used are based on long-term studies within the Health Protection Agency Laboratory of Enteric Pathogens. Ciprofloxacin Etest (AB Biodisk, Solna, Sweden) results were interpreted according to manufacturer’s procedures. A total of 45 *Salmonella* spp. strains were tested. Screening for *qnr* genes by multiplex PCR identified 37 isolates with *qnrS* and 2 carrying *qnrB* variants (Table) (*4*). However, the *qnrB* primer pair in this multiplex did not fully match all *qnrB* gene variants. PCR and sequencing using primers FQ1 and FQ2 (*5*) and *qnrS-F* and *qnrS-R* (*1*), were used to identify specific *qnrB* and *qnrS* gene variants.

**Table:** Isolates of *Salmonella enterica* with plasmid-mediated *qnr* genes, United Kingdom, October 2006–April 2007

*Salmonella s*erotype	Phage type*	No. isolates	VNTR profile†	Ciprofloxacin MIC (μg/mL)‡	Additional resistance to antimicrobial drugs§	*qnr* identified
Corvallis	–	1	–	0.25	S, Su, T	*qnrS1*
Corvallis	–	2	–	0.38	S, Su, T	*qnrS1*
Corvallis	–	1	–	1.0	S, Su, T ,Cf	*qnrS1*
Corvallis	–	1	–	0.25	None	*qnrS1*
Corvallis	–	1	–	0.38	None	*qnrS1*
Schwarzengrund	–	1	–	0.25	T	*qnrB5*
Typhimurium	DT120	4	1–6-0–0-3	0.38	S, Su, T	*qnrS1*
Typhimurium	DT120	3	1–6-0–0-3	0.50	S, Su, T	*qnrS1*
Typhimurium	DT120	3	1–4-0–0-3	0.38	S, Su, T	*qnrS1*
Typhimurium	DT120	1	1–4-0–0-3	0.50	S, Su, T	*qnrS1*
Typhimurium	DT120	1	1–4-0–0-3	0.38	None	*qnrS1*
Typhimurium	DT120	1	1–5-0–0-3	0.38	S, Su, T	*qnrS1*
Typhimurium	DT193	1	1–6-0–0-3	0.50	S, Su, T	*qnrS1*
Typhimurium	DT193	1	1–4-0–0-3	0.38	C, S, Su, Sp, T, Tm	*qnrS1*
Typhimurium	DT193	1	1–4-0–0-3	0.38	S, Su, T	*qnrS1*
Typhimurium	DT193	2	1–5-0–0-3	0.38	S, Su, T	*qnrS1*
Typhimurium	DT193	1	1–4-0–0-3	0.50	A, Su	*qnrS1*
Typhimurium	49b	1	1–4-19–1-3	0.25	A, G, Ne, K, S, Su, Sp, T, Tm, Ak, Cx, Cr, Cf, Cn, Ct	*qnrB2*
Typhimurium	NC	1	1–4-0–0-3	0.25	S, Su, T	*qnrS1*
Typhimurium	UT	1	3–8-19–1-2	>32	A, C, G, S, Su, Sp, T, Tm, Fu, Nx	*qnrS1*
Virchow	43	5	–	1.0	A, Fu, Nx	*qnrS1*
Virchow	43	2	–	1.5	A, Fu, Nx	*qnrS1*
Virchow	25a	1	–	0.75	Tm	*qnrS1*
Virchow	11	1	–	1.0	A, Fu, Nx	*qnrS1*
Virchow	NC	1	–	1.5	A, C, G, Ne, K, S, Su, Sp, T, Tm, Fu, Nx, Cx, Cr, Cf, Cn, Ct	*qnrS1*

*DT, definitive type; NC, does not conform to a recognized pattern; UT, untypeable. †VNTR, variable number tandem repeat. Loci of the VNTR profiles are presented in the following order: STTR9-STTR5-STTR6-STTR10pl-STTR3. The number 0 in the VNTR profile represents cases with no amplification of PCR product. ‡Determined by Etest. §Antimicrobial drugs (breakpoint final concentrations): S, streptomycin (16 mg/L); Su, sulfonamide (64 mg/L); T, tetracycline (8 mg/L); Cf, cefuroxime (16 mg/L); C, chloramphenicol (8 mg/L); Sp, spectinomycin (64 mg/L); Tm, trimethoprim (2 mg/L); A, ampicillin (8 mg/L]; G, gentamicin (4 mg/L); Ne, neomycin (8 mg/L) K, kanamycin (8 mg/L); Ak, amikacin (4 mg/L); Cx, cefalexin (16 mg/L); Cr, cefradine (16 mg/L); Cn, ceftriaxone (1 mg/L); Ct, cefotaxime (1 mg/L); Fu, furazolidone (8 mg/L); Nx, nalidixic acid (16 mg/L).

The *qnrS1*-positive salmonellae belong to serotypes Typhimurium (21 isolates), Virchow (10), and Corvallis (6). Most *S*. Typhimurium isolates were either definitive phage type 120 or 193, and most *S*. Virchow isolates were phage type 43 (Table). Thirteen *qnrS1*-positive isolates were from patients who reported recent travel to Egypt, India, Malaysia, Morocco, Thailand, or an undisclosed destination.

Twelve isolates from patients who had not traveled abroad were assumed to be from UK-acquired infections. *S*. Virchow isolates had been associated with cooked chicken from Thailand (*1*), and *qnrS1* has recently been described in *S*. Corvallis strains from humans in Denmark or isolated in Thailand from humans, chicken, pork, and beef (*3*). Comparison of pulsed-field gel electrophoresis patterns and resistance phenotypes of *qnrS1*-positive *S*. Corvallis strains identified common types, suggesting that some UK patients may have acquired *S*. Corvallis from chicken from Thailand.

Thirteen isolates showed resistance to ceftriaxone, cefotaxime, or ampicillin. Plasmids with *qnr* genes have been found to co-transfer TEM, SHV, and CTX-M genes (*1**,**5**,**6*). Co-transmission of fluoroquinolone and β-lactamase resistance is clinically important because co-selection of resistance by use of either drug may occur.

Twenty-one *qnrS1*-positive *S*. Typhimurium were subtyped by variable number tandem repeat (VNTR) analysis to determine whether the increase was caused by spread of >1 distinct strains (*7*). Twenty isolates produced 1 of 3 related profiles (loci of VNTR profiles are ordered STTR9-STTR5-STTR6-STTR10pl-STTR3): 1–4-0–0-3, 9 isolates; 1–5-0–0-3, 3 isolates; or 1–6-0–0-3, 8 isolates. Alleles 4 and 5, and 5 and 6 at locus STTR5 only differed by an extra 6-bp repeat, which suggests a clonal relationship between the *qnrS1*-positive *S*. Typhimurium in this study (Table) (*8*). *S*. Typhimurium isolates with the 1–6-0–0-3 profile have been isolated from tourists returning from Asia (*7*), which suggests that the UK *qnrS1*-positive *S*. Typhimurium isolates have originated in the Far East.

These findings show increased occurrence of *qnr* genes, particularly *qnrS1*, in nontyphoidal salmonellae in the United Kingdom. These data are in contrast to those of recent studies in the United States and France, which show low incidences of *qnrS* genes in larger strain collections (*9**,**10*). The *qnr* phenotype is in contrast to resistance mediated by mutations in the topoisomerase genes whereby 1 mutation confers low-level resistance to fluoroquinolones and full resistance to nalidixic acid. Our previous study demonstrated that *qnrS1* was sufficient to cause decreased susceptibility to ciprofloxacin in the absence of mutations in *gyrA* (*1*). In this study, a *qnr* gene was sufficient to increase the ciprofloxacin MIC to 0.38–0.75 μg/mL. In addition, a *qnr* gene contributed to high-level ciprofloxacin resistance in 10 isolates, thereby potentially jeopardizing first-line treatment of vulnerable patient groups with ciprofloxacin.

## References

[1] Hopkins KL, Wootton L, Day M, Threlfall EJ. Plasmid-mediated quinolone resistance determinant *qnrS1* found in *Salmonella enterica* strains isolated in the UK. J Antimicrob Chemother. 2007;59:1071–5. 10.1093/jac/dkm081 17434878

[2] Cavaco LM, Hansen DS, Friis-Møller A, Aarestrup FM, Hasman H, Frimodt-Møller N. First detection of plasmid-mediated quinolone resistance (*qnrA* and *qnrS*) in *Escherichia coli* strains isolated from humans in Scandinavia. J Antimicrob Chemother. 2007;59:804–5. 10.1093/jac/dkl554 17284539

[3] Cavaco LM, Hendriksen RS, Aarestrup FM. Plasmid-mediated quinolone resistance determinant *qnrS1* detected in *Salmonella enterica* serovar Corvallis strains isolated in Denmark and Thailand. J Antimicrob Chemother. 2007;60:704–6. 10.1093/jac/dkm261 17635876

[4] Robicsek A, Strahilevitz J, Sahm DF, Jacoby GA, Hooper DC. *qnr* prevalence in ceftazidime-resistant *Enterobacteriaceae* isolates from the United States. Antimicrob Agents Chemother. 2006;50:2872–4. 10.1128/AAC.01647-05 16870791 PMC1538681

[5] Jacoby GA, Walsh KE, Mills DM, Walker VJ, Oh H, Robicsek A, *qnrB*, another plasmid-mediated gene for quinolone resistance. Antimicrob Agents Chemother. 2006;50:1178–82. 10.1128/AAC.50.4.1178-1182.2006 16569827 PMC1426915

[6] Jacoby GA, Chow N, Waites KB. Prevalence of plasmid-mediated quinolone resistance. Antimicrob Agents Chemother. 2003;47:559–62. 10.1128/AAC.47.2.559-562.2003 12543659 PMC151764

[7] Lindstedt BA, Torpdahl M, Nielsen EM, Vardund T, Aas L, Kapperud G. Harmonization of the multiple-locus variable-number tandem repeat analysis method between Denmark and Norway for typing *Salmonella* Typhimurium isolates and closer examination of the VNTR loci. J Appl Microbiol. 2007;102:728–35. 10.1111/j.1365-2672.2006.03134.x 17309622

[8] Hopkins KL, Maguire C, Best E, Liebana E, Threlfall EJ. Stability of multiple-locus variable-number tandem repeats in *Salmonella enterica* serovar Typhimurium. J Clin Microbiol. 2007;45:3058–61. 10.1128/JCM.00715-07 17609320 PMC2045244

[9] Gay K, Robicsek A, Strahilevitz J, Park CH, Jacoby G, Barrett TJ, Plasmid-mediated quinolone resistance in non-Typhi serotypes of *Salmonella enterica.* Clin Infect Dis. 2006;43:297–304. 10.1086/505397 16804843

[10] Cattoir V, Weill FX, Poirel L, Fabre L, Soussy CJ, Nordmann P. Prevalence of *qnr* genes in *Salmonella* in France. J Antimicrob Chemother. 2007;59:751–4. 10.1093/jac/dkl547 17307773

